# Identification and characterization of a NBS–LRR class resistance gene analog in *Pistacia atlantica* subsp. Kurdica

**Published:** 2014-09

**Authors:** Bahman Bahramnejad

**Affiliations:** Department of Agricultural Biotechnology, Faculty of Agriculture, University of Kurdistan, Sanandaj, Iran

**Keywords:** *Pistacia atlantica* subsp. Kurdica, Resistance gene analog (RGA), cloning

## Abstract

*P. atlantica* subsp. Kurdica, with the local name of Baneh, is a wild medicinal plant which grows in Kurdistan, Iran. The identification of resistance gene analogs holds great promise for the development of resistant cultivars. A PCR approach with degenerate primers designed according to conserved NBS-LRR (nucleotide binding site-leucine rich repeat) regions of known disease-resistance (R) genes was used to amplify and clone homologous sequences from *P. atlantica* subsp. Kurdica. A DNA fragment of the expected 500-bp size was amplified. The nucleotide sequence of this amplicon was obtained through sequencing and the predicted amino acid sequence compared to the amino acid sequences of known R-genes revealed significant sequence similarity. Alignment of the deduced amino acid sequence of *P. atlantica* subsp. Kurdica resistance gene analog (RGA) showed strong identity, ranging from 68% to 77%, to the non-toll interleukin receptor (non-TIR) R-gene subfamily from other plants. A P-loop motif (GMMGGEGKTT), a conserved and hydrophobic motif GLPLAL, a kinase-2a motif (LLVLDDV), when replaced by IAVFDDI in* PAKRGA1* and a kinase-3a (FGPGSRIII) were presented in all RGA. A phylogenetic tree, based on the deduced amino-acid sequences of *PAKRGA1* and RGAs from different species indicated that they were separated in two clusters, *PAKRGA1* being on cluster II. The isolated NBS analogs can be eventually used as guidelines to isolate numerous R-genes in Pistachio.

## INTRODUCTION

Wild pistachio (*Pistacia*) is a genus of the tropical Anacardiaceae family plants. Three *Pistacia* species grow naturally in Iran, including *P. vera* Linnaeus, *P. khinjuk* and *P. atlantica* Desf. A large population of wild *P. atlantica* subsp. Kurdica trees, locally known as Baneh, grow across Kurdistan province, Iran. *P. atlantica* subsp. Kurdica is used for medicine, food and industrial purposes. Native people grind and mix baneh nuts with other nuts. The gum is also used in the production of chewing gum [1]. Biological effects of gum compounds, including anti-atherogenic, hypoglycemic, anti-inflammatory, antipyretic, antifungal, antimicrobial, anti-viral, anti-insecticide and anticancer activities of the *Pistacia* species have all been studied previously [2]. In addition, *P. atlantica* rootstock has been found to be resistant to nematodes as well as drought stress, thus a suitable rootstock for dry regions [3-5].

Plants have innate immune responses which involve the plant’s resistance gene (R-gene). This gene plays a key role in recognizing proteins  expressed by specific avirulence (Avr) genes of pathogens, thus defending the plant against attacks from fungi, oomycetes, bacteria, viruses, insect pests, and nematodes. Such genes are classified into five categories based on their predicted protein [6, 7] Five diverse classes have also been identified based on specific conserved functional domains [8]. The NBS (nucleotide-binding site)-LRR (leucine-rich repeat) family is the most abundant class showing resistance to a number of pathogens. The NBS domain located at the N-terminal end contains several highly conserved motifs such as the P-loop/kinase 1, kinase 2 and kinase 3-a and hydrophobic GLPL. P-loop (phosphate-binding loop) is a motif in ATP- and GTP-binding proteins involved with an ATP synthase β subunit, ras protein, ribosomal elongation factor, and adenylate kinase with molecular switches [9]. 

The PCR approach for cloning NBS-LRR genes with degenerate primers is based on conserved amino acid motifs of a known NBS-LRR gene. This method has been widely used to find NBS-encoding disease resistant gene analogs (RGAs) from a variety of species, including *Arabidopsis thaliana *[3], tomatoes [10], soybeans [11], strawberries [12], poplar [13], and a wild species of peach (*Prunus kansuensis*) [14]. 

The aim of this study was to isolate a number of RGAs from *P. atlantica *subsp. Kurdica in order to facilitate the functional cloning of R genes. A pair of degenerate oligonucleotide primers was used based on the NBS domain of resistance genes and a new resistance gene analog was obtained from the of *P. atlantica *subsp. KurdicaKurdica by PCR analysis. Finally, an NBS-LRR class of RGAs obtained from of *P. atlantica *subsp. Kurdica was compared with other known resistance gene sequences.

## MATERIALS AND METHODS

**Plant materials and DNA extraction: **Seeds of *P. atlantica *subsp. Kurdica were collected from female trees in Kamyaran city, south of Kurdistan province, soaked in water and shaken for two days at 150 rpm. The surface layer was then gently removed. Seed scarification was done by soaking in concentrated sulfuric acid (30N) in a glass container and gently stirring for 10 minutes. When the seed coat was modified (thinned), the seeds were removed, washed with distillated water and sown. A month after the seedlings emerged and were about 10 cm high, small leaves were used for DNA extraction. Total genomic DNA was isolated from the combined leaves of five *P. atlantica* subsp. Kurdica plants using the protocol described by Doyle and Doyle[15] (1990) with required modifications. The quality and quantity of the DNA were determined using a WPA (Biochrom) spectrophotometer and 1% agarose gel. 

**Cloning and sequencing of NBS-LRR-type sequences: **R-gene-specific degenerate oligonucleotide primers, previously used in other taxa [16], were selected. A forward primer RGAF1 was designed in sense direction, corresponding to the amino acid sequence GMGGVGKT of the NBS motif: 5′-GGNATGGGNGGNGTNGGNA A(A/G)AC-3′, and the reverse primer RGAR1 was based on the amino acid sequence GLPLALKV of the membrane-spanning motif in anti-sense direction: 5′-AC(T/C)TTN A(A/G) NGCNA(A/G)NGGNA(A/G)NCC-3′. The reverse primer was based on motif (GLPLAL) from the *N*, *L6 *and *RPS2 *genes of the NBS-LRR class specific against pathogens. The primers were designed to obtain around 500-bp PCR products upon amplification. PCR was carried in a 25-μL reaction mixture with 20 ng template DNA, 200 nM of each forward and reverse primer, 200 μM of each dATP, dCTP, dGTP, and dTTP, 1X PCR buffers, 15 mM MgCl2 and 1 U *Taq *DNA polymerase (Vivantis). PCR was performed in an icycler thermal cycler (BIORAD) using the following cyclic conditions: initial denaturation at 94°C for 5 min followed by 42 cycles each consisting of DNA denaturation at 94°C for 1 min, primer annealing at 53°C for 1 min and primer extension at 72°C for 1 min and a final extension for 7 min. PCR products were subjected to electrophoresis on a 1.5% agarose gel in a 0.5X TAE buffer, at 60 V for 1 h. Gel photographs were taken for recording and analysis purposes using a gel documentation system (Biorad). PCR products were separated on 1.0% agarose gels and the expected fragments were purified from the gels using a Nucleic Acid Extraction kit (Vivantis). Purified DNA concentrations were determined by a spectrophotometer. DNA fragments were then ligated into the TA vector using a TA cloning kit (Fermentas) and transformed into competent cells of the *Escherichia coli* *DH5a* strain. Positive clones were identified by colony PCR and independent sequences per clone were obtained from a commercial sequencing service (Bioneer Inc. Bioneer Corporation).

**Bioinformatic analysis: **Sequenced fragments provided highly accurate DNA sequence information and were submitted to sequence analysis. To search for characteristic motifs of resistance proteins, nucleotide sequences were translated and corresponding amino acid sequences were aligned with NBS domains encoded by cloned R-genes using CLUSTALW software [17]. To identify resistance gene analogues as well as other homologous sequences in the database, a homology search was performed using the default settings of BLASTp with the non-redundant GenBank database (http://www.ncbi.nlm.nih.gov). Amino acid sequences from resistance genes of other plant species were added to the set of NBS sequences, and cluster analysis was carried out using the MEGA package based on the neighbor-joining method [18].

## RESULTS AND DISCUSSION

**Figure 1:**
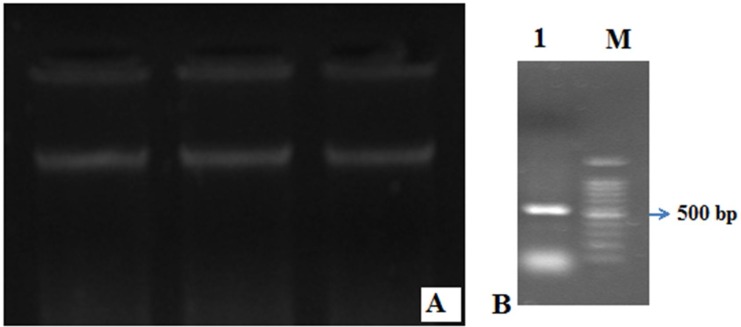
Genomic DNA of *Pistacia atlantica subsp. kurdica* and PCR amplification of *PAKRGA1*. A) Genomic DNA of three different sample of young seedlings. B) PCR amplification of *PAKRGA1 *using the combined degenerate primers designed according to the conserved NBS-LRR motif of several plant R genes. M is the 2-kb DNA ladder, Line 1 is a *P. atlantica subsp. kurdica* PCR product

Using the two R-gene specific degenerate primers, an RGA candidate was isolated from *P*. *atlantic* subsp. Kurdica leaves. Genomic DNA was efficiently isolated from leaves of young *P. atlantica* subsp Kurdica seedlings (Fig. 1A). PCR amplification with genomic DNA resulted in the production of amplicons with predicted sizes of about 500 bp, based on previously published RGA sequences (Fig. 1B). Amplicons were cloned and five colonies were sequenced. Following homology searches with BLASTX algorithms, the sequenced fragment showed significant homology to the NBS domain of known R-genes or the RGCs cloned from other plant species (Table 1) and was therefore, designated as *PAKRGA1*. These *PAKRGA1* sequences showed a high level of sequence identity to comparable regions of disease resistance genes published in GenBank, supported by low e-values. BLASTP searches of deduced amino acid sequences of the PAKRGA1 revealed the presence of an NBS domain and significant homology to well-characterized R genes from angiosperms.

**Table 1:** Sequence homology comparisons of *PAKRGA1*with highest similarity sequences, expected value and identity percentage

**Accession**	**Description**	**E- value**	**Identities**
GU550694.1	Citrus reticulata x Citrus trifoliata clone F29 nucleotide binding site protein gene, partial cds	2e-105	77%
HQ238252.1	Citrus reticulata x Citrus trifoliata clone 20.F2.3 resistance protein-like protein gene, partial cds	4e-83	76%
HQ238253.1	Citrus reticulata x Citrus trifoliata clone 20.F2.4 resistance protein-like pseudogene, partial sequence	6e-68	74%
XM_004298550.1	PREDICTED: Fragaria vesca subsp. vesca TMV resistance protein N-like (LOC101310990), mRNA	3e-52	69%
XM_002520135.1	Ricinus communis leucine-rich repeat-containing protein, putative, mRNA	6e-49	69%
XM_002268288.2	PREDICTED: Vitis vinifera protein SUPPRESSOR OF npr1-1, CONSTITUTIVE 1-like (LOC100241580), mRNA	7e-48	69%
XM_002321458.1	Populus trichocarpa tir-nbs-lrr resistance protein, mRNA	4e-45	69%
EF653094.1	Platanus x acerifolia isolate F11M3.68 NBS-containing resistance-like protein gene, partial cds	4e-45	70%
FJ789948.1	Rosa hybrid cultivar isolate F3P1-2H NBS-LRR resistance protein gene, partial cds	5e-43	68%
JN990341.1	Rubus glaucus clone C92_P1_P2 putative TIR-NBS-LRR disease resistance protein gene, partial cds	5e-37	73%
FJ185475.1	Corylus avellana isolate cav.w.F10.11 NBS-containing resistance-like protein gene, partial cds	6e-36	67%
XM_002321460.1	Populus trichocarpa tir-nbs-lrr resistance protein, mRNA	1e-31	67%
XM_003628529.1	Medicago truncatula Tir-nbs-lrr resistance protein (MTR_8g062130) mRNA, complete cds	7e-29	69%
DQ644288.1	Malus x domestica clone ABHA006062CT putative NBS-LRR disease resistance protein gene, partial cds	2e-17	68%
	

**Figure 2:**
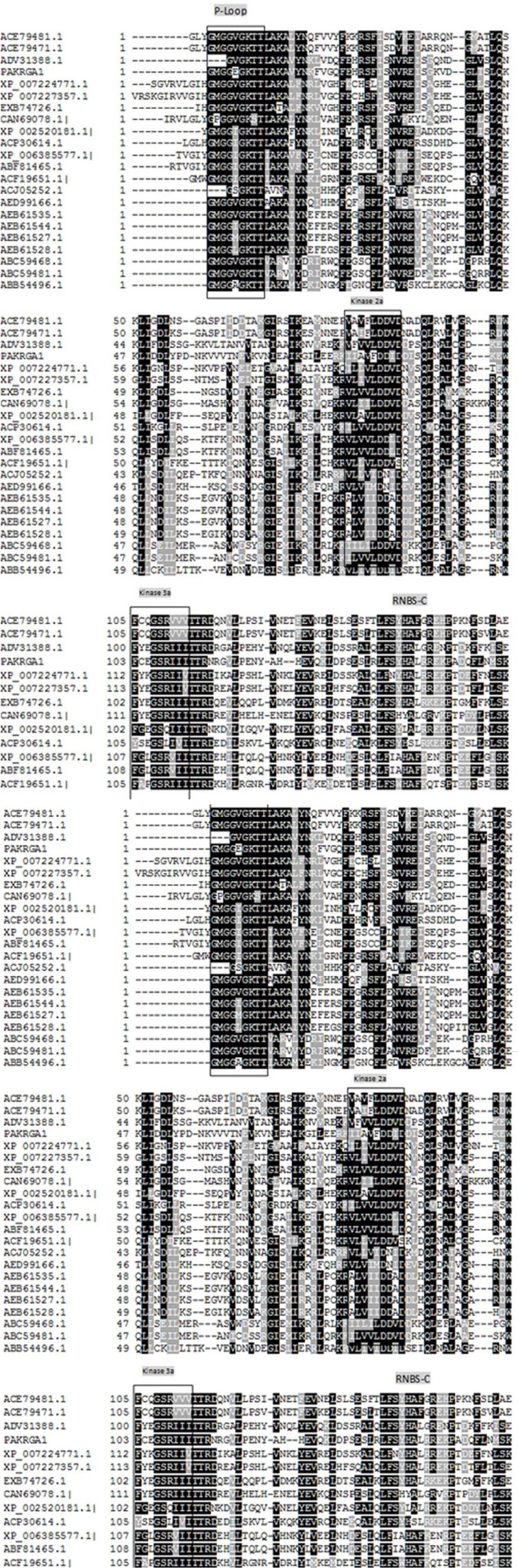
Multiple alignments of the consensus amino acid sequences of the 21 RGAs and NBS domain of R-genes along with PAKRGA1 of* Pistacia atlantica subsp. kurdica* constructed with Clustal W. Conserved motifs are numbered as in Lescot et al. (2004); P-loop, Kinase-2a, Kinase-3a, RNBS-C and GLPL motifs

Multiple alignments of the deduced amino acid sequences of PAKRGA1 along with the selected RGA sequences from other plants revealed the presence of the conserved resistance P-loop motif (GMGGVGKT) (Fig. 2). In addition, kinase-2a (LLVLDDV), kinase-3a (FGPGSR) and GLPL motifs were present in PAKRGA1. The analysis showed that PAKRGA1 motifs’ P-loop, kinase-3a and GLPL were highly conserved. Nevertheless, the kinase-2a motif was relatively less conserved and was replaced by IAVFDDI. Analyses of nucleotide polymorphisms, *P. atlantica* subsp. Kurdica diversity and other RGAs showed them to be highly conserved at the P-loop and comparatively more conserved at Kinase-2a, and Kinase-3a and GLPL motifs than other sequence parts. This high conservation at the P-loop and GLPL motifs of RGAs might be due to degenerate primer sequences.

**Figure 3:**
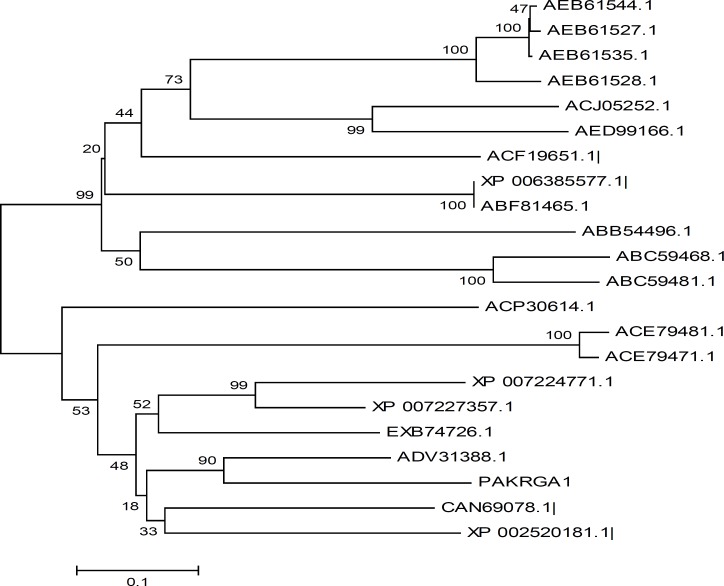
Consensus tree of NBS encoding RGAs in the* Pistacia atlantica subsp. kurdica* constructed by phylogenetic analysis using the clustalW program. ADV31388.1 (*Citrus reticulata x Citrus trifoliate), *ACE79481.1 (*Nicotiana sylvestris*), ACE79471.1 (*Nicotiana tabacum*), XP_002520181.1 (* Ricinus communis*), EXB74726.1 (*Morus notabilis*), XP_007224771.1 (*Prunus persica*), XP_007227357.1 (*Prunus persica*), CAN69078.1 (*Vitis vinifera*), ACP30614.1 (*Brassica rapa subsp.* Pekinensis), AED99166.1 (*Malus baccata*), ABC59468.1 ( *Populus tomentosa x Populus bolleana) x Populus tomentosa var. *truncate), ABC59481.1 *Populus tomentosa x Populus bolleana) x Populus tomentosa var. *truncate), XP_006385577.1 (*Populus trichocarpa*), ABF81465.1 (*Populus trichocarpa*), ABB54496.1 ( *Ipomoea batatas*) ACF19651.1 (* Medicago sativa*), ACJ05252.1 ( *Pyrus pyrifolia*), AEB61535.1 (*Prunus persica*), PAKRGA1 (*Pistacia atlantica* subsp. Kurdica), AEB61528.1 ( *Prunus persica*), AEB61527.1 ( *Prunus persica*), AEB61544.1 ( *Prunus persica*

To estimate phylogenetic relationships among *PAKRGA1* and other sequences of known plant NBS encoding R genes, a neighbor-joining phylogenetic tree was constructed from which the genetic divergence between RGA sequences was observed. The sequences were classified into two groups. *PAKRGA1* were classified in class II along with RGAs; ADV31388.1 (*Citrus reticulata x Citrus trifoliate), *ACE79481.1 (*Nicotiana sylvestris*), ACE79471.1 (*Nicotiana tabacum*), XP_002520181.1 (* Ricinus communis*), EXB74726.1 (*Morus notabilis*), XP_007224771.1 (*Prunus persica*), XP_007227357.1 (*Prunus persica*), CAN69078.1 (*Vitis vinifera*), ACP30614.1 (*Brassica rapa subsp.* Pekinensis) (Fig. 3). In the other group, RGAs AED99166.1 (*Malus baccata*), ABC59468.1 (*Populus tomentosa x Populus bolleana) x Populus tomentosa var. *truncate), ABC59481.1 *Populus tomentosa x Populus bolleana) x Populus tomentosa var. *truncate), XP_006385577.1 (*Populus trichocarpa*), ABF81465.1 (*Populus trichocarpa*), ABB54496.1 (*Ipomoea batatas*) ACF19651.1 (*Medicago sativa*), ACJ05252.1 (*Pyrus pyrifolia*), AEB61535.1 (*Prunus persica*), AEB61528.1 (*Prunus persica*), AEB61527.1 (*Prunus persica*), AEB61544.1 ( *Prunus persica*) were classified.

PCR amplification with degenerate oligonucleotide primers is a sensitive and efficient method of cloning RGAs, which are potential candidates for functional resistance genes [11]. In the present study, we isolated a genomic RGA of the NBS-LRR type from *P. atlantica* subsp. Kurdica. The PCR derived sequence was identified as RGA based on high sequence identities to known R genes/RGAs from other species, presence of conserved motifs characteristic of NBS-LRR R genes and uninterrupted ORFs with considerable length  [19] . Percentage identity and the e (expected) value of *PAKRGA1* to RGAs from other plant species ranged from 39% to 62% and 4e-39 to 9e-30, respectively. Using a similar approach, NBS sequences identified in other plant species have also showed identity ranges comparable to RGAs. The *PAKRGA1* P-loop conserved domain was very similar to other RGAs, while kinase domains were quite variable. The last residue of the kinase-2 motif can be used to predict a subclass of NBS–LRR R-genes with 95% accuracy [20]. A tryptophan residue (W) is expected at the end of the kinase-2 motif in non-TIR NBS-LRR sequences while an aspartic acid (D) or asparagine (N) residue is expected for TIR NBS–LRR sequences. Using this criterion, we showed that *PAKRGA1* belonged to the TIR NBS–LRR subclass.

The phylogenetic analysis of NBS analogs identified their group based on similarity. The phylogenetic tree based on neighbor joining using the percentage of identity of the deduced amino acid sequences of *P. atalatica *subsp. Kurdica and other species identified their relatedness with known R-genes. Similar to previous reports [16], the phenetic tree classified the RGAs into two classes. However, the isolation, sequencing and analysis of more *Pistacia *NBS analogs are required to gain better knowledge about the RGAs in *Pistacia* and to draw further conclusions.

Four important *Pistacia* species, *P. vera* L., *P. khinjuk* stocks *P. eurycarpa* Yalt. (*P. atlantica* subsp. Kurdica Zoh.) and *P. atlantica* Dsef. originate from Iran [21]. *Pistacia atlantica* and *P. khinjuk* are the rootstocks most resistant to root-knot nematode and drought. It is thus possible that *P. atlantica* subsp. Kurdica have large numbers of resistance genes. Using identified *P. atlantica *subsp. Kurdica RGA, different primer sets can be designed to analyze *Pistacia’s* wild relatives*,* to discover their novel genomic resources and to improve their genetic composition**.**
